# New models and online calculator for predicting non-sentinel lymph node status in sentinel lymph node positive breast cancer patients

**DOI:** 10.1186/1471-2407-8-66

**Published:** 2008-03-04

**Authors:** Holbrook E Kohrt, Richard A Olshen, Honnie R Bermas, William H Goodson, Douglas J Wood, Solomon Henry, Robert V Rouse, Lisa Bailey, Vicki J Philben, Frederick M Dirbas, Jocelyn J Dunn, Denise L Johnson, Irene L Wapnir, Robert W Carlson, Frank E Stockdale, Nora M Hansen, Stefanie S Jeffrey, The Bay Area SLN Study

**Affiliations:** 1 Department of Medicine, Stanford University School of Medicine, Stanford, CA, USA; 2 Department of Health Research and Policy-Biostatistics, Stanford University School of Medicine, Stanford, CA, USA; 3 Departments of Statistics and Electrical Engineering, Stanford University, Stanford, CA, USA; 4 Department of Surgery, Northwestern University Feinberg School of Medicine and Lynn Sage Comprehensive Breast Center, Northwestern Memorial Hospital, Chicago, IL, USA; 5 Department of Surgery, California Pacific Medical Center, San Francisco, CA, USA; 6 Department of Pathology, Stanford University School of Medicine, Stanford, CA, USA; 7 Department of Surgery, Alta Bates Summit Medical Center, Berkeley, CA, USA; 8 Department of Surgery, Mercy Medical Center, Redding, CA, USA; 9 Department of Surgery, Stanford University School of Medicine, Stanford, CA, USA

## Abstract

**Background:**

Current practice is to perform a completion axillary lymph node dissection (ALND) for breast cancer patients with tumor-involved sentinel lymph nodes (SLNs), although fewer than half will have non-sentinel node (NSLN) metastasis. Our goal was to develop new models to quantify the risk of NSLN metastasis in SLN-positive patients and to compare predictive capabilities to another widely used model.

**Methods:**

We constructed three models to predict NSLN status: recursive partitioning with receiver operating characteristic curves (RP-ROC), boosted Classification and Regression Trees (CART), and multivariate logistic regression (MLR) informed by CART. Data were compiled from a multicenter Northern California and Oregon database of 784 patients who prospectively underwent SLN biopsy and completion ALND. We compared the predictive abilities of our best model and the Memorial Sloan-Kettering Breast Cancer Nomogram (Nomogram) in our dataset and an independent dataset from Northwestern University.

**Results:**

285 patients had positive SLNs, of which 213 had known angiolymphatic invasion status and 171 had complete pathologic data including hormone receptor status. 264 (93%) patients had limited SLN disease (micrometastasis, 70%, or isolated tumor cells, 23%). 101 (35%) of all SLN-positive patients had tumor-involved NSLNs. Three variables (tumor size, angiolymphatic invasion, and SLN metastasis size) predicted risk in all our models. RP-ROC and boosted CART stratified patients into four risk levels. MLR informed by CART was most accurate. Using two composite predictors calculated from three variables, MLR informed by CART was more accurate than the Nomogram computed using eight predictors. In our dataset, area under ROC curve (AUC) was 0.83/0.85 for MLR (n = 213/n = 171) and 0.77 for Nomogram (n = 171). When applied to an independent dataset (n = 77), AUC was 0.74 for our model and 0.62 for Nomogram. The composite predictors in our model were the product of angiolymphatic invasion and size of SLN metastasis, and the product of tumor size and square of SLN metastasis size.

**Conclusion:**

We present a new model developed from a community-based SLN database that uses only three rather than eight variables to achieve higher accuracy than the Nomogram for predicting NSLN status in two different datasets.

## Background

Current practice guidelines recommend a completion axillary lymph node dissection for breast cancer patients whose SLN contains metastatic tumor [1-3]. The risk of morbidity that accompanies completion ALND seems justified for patients with NSLN metastases, because they would undergo excision of residual cancer [4]. However, 50 to 65% of patients with tumor-involved SLNs do not have additional nodal metastasis [5,6]. For them, ALND offers no clear therapeutic benefit, provides no further information for staging, and increases the cost of medical care. Further, completion ALND is associated with substantial morbidity affecting up to 39% of patients, with a nearly three-fold increased risk of lymphedema or regional sensory loss [7-9]. Identifying SLN-positive patients without NSLN metastases who could forgo completion ALND would improve the quality of life and reduce costs for the majority of women with new diagnoses of breast cancer.

Previous investigations have not identified predictors of NSLN status with accuracy sufficient to change clinical practice. This failure may be due to limited sample sizes or single institution studies [5,6,10]. The majority of prior investigations include sample sizes of less than two hundred subjects, with the challenges of dealing with small sample sizes leading to decreased predictive accuracy when applied to the general population [5,6,10-12]. However in 2003 Van Zee *et al. *proposed a nomogram to predict risk of NSLN metastasis based on an accrued population of 1075 cases of primary invasive breast cancer [13]. The Memorial Sloan-Kettering Cancer Center (MSKCC) Breast Cancer Nomogram (Nomogram) has since been successfully applied internationally and become the most commonly used predictive model for NSLN involvement [14]. Use of a predictive nomogram has been shown to be superior to expert opinion, to improve clinical decision making, and to be partially responsible for the decreasing frequency of ALNDs performed [15,16]. However, use of the Nomogram is limited by its complexity, and inability to be applied if not all patient characteristics are known [17]. Although the Nomogram was based on a large sample size, its reported predictive accuracy and its generalizability to patient populations with dissimilar tumor characteristics or to non-academic, non-quaternary care hospitals has been questioned [17-19].

Our goal was to identify characteristics of patients and their tumors that predict NSLN status within the Bay Area SLN Database, comprised of diverse patient populations from one academic and 15 community-based medical centers in Northern California and Oregon. We constructed three new models and contrasted their performance with the Nomogram. We provide a model that has simpler input than the Nomogram and shows higher accuracy for our diverse patient population and for another population of SLN-positive patients with different patient characteristics from Northwestern University. We have created an internet-based calculator, the Stanford Online Calculator, for validation testing and clinical application.

## Methods

### Study patients

The *Bay Area SLN Study for Detection of Axillary Metastasis in Breast Cancer *is a multi-institutional collaboration involving 16 institutions in the Greater Bay Area of Northern California and Oregon, of which 15 are community hospitals. A total of 1,040 patients underwent SLN biopsy for biopsy-proven breast cancer between 1996 and 2002. After excluding 256 patients (criteria shown in Additional file 1), we analyzed 784 prospectively accrued subjects with primary invasive breast carcinoma and clinically negative axilla who underwent SLN biopsy with completion axillary lymph node dissection. 285 (36.4%) had tumor-involved SLNs. Among the 285 SLN-positive patients, 213 had pathologic information regarding presence or absence of angiolymphatic invasion (lymphovascular invasion, LVI); 171 patients had complete pathologic information on both angiolymphatic invasion and hormone receptor status. The *Northwestern *test dataset was compiled by chart review of all patients who underwent a SLN biopsy at Northwestern Memorial Hospital in Chicago, IL, between 2002 and 2006. It is comprised of 77 consecutively identified sentinel node positive patients with invasive breast cancer who underwent completion ALND and had complete pathologic information on tumor type, tumor size, tumor grade, hormone receptor status, HER2/*neu *status, angiolymphatic invasion status, number of nodes removed, and size of sentinel node metastases. Inclusion and exclusion criteria are similar to that outlined for the Stanford patients in Additional file 1. The Northwestern database was compiled by physicians not involved in generation of the predictive models. The Bay Area SLN study was performed under a protocol approved by the Stanford University Administrative Panel on Human Subjects in Medical Research and the Institutional Review Boards of each participating institution. An independent protocol was approved by the Institutional Review Board of Northwestern University for retrospective chart review and data collection to test the Stanford Online Calculator and MSKCC Nomogram.

### SLN biopsy and pathological evaluation

SLN biopsy has been described previously [20]. The SLN was identified using peritumoral injection of 1% isosulfan blue dye, filtered ^99m^Tc sulfur colloid radioactive tracer, or both, as decided by the operating surgeon. All lymph nodes that were blue and/or focally radioactive and/or suspicious by intraoperative palpation were denoted SLNs. All SLNs were evaluated by step-sectioning with hematoxylin and eosin (H&E) staining; in the Bay Area SLN study, SLNs without metastasis detectable by H&E underwent staining by immunohistochemistry (IHC) [21]. IHC was performed on at least four levels of the SLN using anti-keratin antibodies AE1 and CAM5.2. One pathologist directed and interpreted IHC studies on every SLN excised at 14 of the 16 participating institutions in the Bay Area SLN Study. NSLNs were evaluated by H&E only, without serial sectioning. In the Northwestern series, negative SLNs did not undergo IHC testing and individual tumor cells or clusters were identified on H&E only.

### Statistical analyses

Thirteen characteristics were studied individually for predicting NSLN status: patient age, tumor histology, tumor size (as a continuous variable and as T size by 6^th ^edition AJCC criteria), tumor grade [22], estrogen receptor (ER) status, progesterone receptor (PR) status, HER2/*neu *status, presence of angiolymphatic invasion, number of SLNs excised, number of positive SLNs, size of nodal metastasis (recorded according to revised 6^th ^edition AJCC criteria) [23], and method of detecting nodal metastasis (H&E or IHC). Univariate testing was done with χ^2 ^statistics and Wilcoxon rank sums. For multivariate analyses, tree-based classification and logistic regression were performed [24,25]. Recognizing that some characteristics can be interdependent, we performed multivariate analyses with two approaches whereby interactions among variables are emphasized: recursive partitioning via receiver operating characteristic (RP-ROC) [26] curves and (boosted) classification and regression trees (CART^®^) [24,27,28].

RP-ROC uses the relationship of sensitivity and specificity to calculate the "best value" of each variable for predicting NSLN status. It then chooses the variable with best value. Successive partitioning permits use of ROC curves to compare predictive accuracy and best cut point on "best selected variable." Partitioning of the population into subgroups continues until only patients with or without NSLN metastases are segregated to the group, or until the putative *p *value of the split exceeds 0.01. RP-ROC was performed as is described in detail by Kraemer [26] (software available from Sierra-Pacific MIRECC [29]).

CART as we applied it uses both cross-validation and voting methods (boosting) to assess the stability and improve the accuracy of the final model [24,27], (software available from Salford Systems, v5 [30]). Splits are chosen by what is termed the Gini criterion, whose goal is to render nodes of the tree as "pure" as possible in terms of positive or negative NSLN status. Boosting is a method designed to focus on "hard to classify" observations. In all classifications, there is dependence on the products by class of priors and costs of misclassification. For all classification trees, mixed priors (an average of equal priors and prevalence-based priors) were used. After surveying eighteen breast surgeons expert in SLN biopsy and not associated with this study, the costs of a false-positive and false-negative NSLN were set at 3 and 10, respectively.

A third technique, multivariate logistic regression (MLR) informed by CART, was performed with variable selection based on paths from the root to the five terminal nodes of unboosted CART [31]. Odds ratios were calculated individually for all terms that were candidates for inclusion in subsequent analyses. Those terms retained were entered into the MLR by forward selection based on the likelihood ratio. Wald statistics and odds ratios were determined for variables significant at putative *p *< 0.01 within the regression model [32]. A cutoff p < 0.01 was chosen in the interest of our ending with a focused, concise, predictive model.

In constructing the predictive models of NSLN status, we used tumor characteristics that were significant by univariate testing (Table 1): tumor size, tumor grade, ER status, PR status, angiolymphatic invasion, size of SLN metastasis, and SLN metastasis identification method. Statistical modeling of NSLN status allowed calculation of both the predictive capacity of significant variables and the critical interactions between and among variables, such as increasing angiolymphatic invasion with increasing tumor size. All models used identical variables, although not identical patients. RP-ROC requires complete data, where no values of features are missing, whereas CART does not. Instead, CART relies on the subtle notion of "surrogate split" [24]. Thus, boosted CART analyses were performed on all 285 SLN-positive patients as well as subsets with more complete information, while RP-ROC and MLR analyses were performed on the 213 patients with complete data for angiolymphatic invasion and on the 171 patients with complete data for angiolymphatic invasion and hormone receptor status.

**Table 1:** Characteristics of NSLN- and NSLN+ cases among SLN+ patients (Bay Area SLN Database).

	**Tumor-free NSLN**				**Tumor-involved NSLN**				*Univariate P *∫	*Multivariate P *∫∫	**%NSLN+ **(NSLN+/SLN+)	**TOTAL SLN+**	
***Patient and Tumor Characteristics***	Number of Pts (n = 184)	*%*	Mean	SEM*	Number of Pts (n = 101)	*%*	Mean	SEM*				Number of Pts (n = 285)	*%*

**Patient Age (years)**			55.8	0.89			53	1.14	0.084	NA			
**Tumor Type**													
Infiltrating Ductal Carcinoma	159	*86*			87	*86*			0.781	NA	35%	246	*86.3*
Invasive Lobular Carcinoma	18	*10*			9	*9*					33%	27	*9.5*
Mixed Carcinoma	6	*3*			5	*5*					45%	11	*3.9*
Tubular Carcinoma	1	*1*			0	*0*					0%	1	*0.4*
**Tumor size (cm)**			2.11	0.1			2.97	0.18	<0.001	<0.001			
**Tumor size (AJCC)**									0.001	0.045			
T1	117	*64*			39	*39*					25%	156	*54.7*
T1a (mic)	1	*1*			0	*0*					0%	1	*0.4*
T1a	6	*3*			2	*2*					25%	8	*2.8*
T1b	19	*10*			3	*3*					14%	22	*7.7*
T1c	91	*49*			34	*34*					27%	125	*43.9*
T2	59	*32*			50	*50*					46%	109	*38.2*
T3	8	*4*			12	*12*					60%	20	*7.0*
**Tumor grade†**									0.001	0.736			
G1: Nottingham combined histologic score 3–5	68	*37*			23	*23*					25%	91	*31.9*
G2: Nottingham combined histologic score 6–7	81	*44*			39	*39*					33%	120	*42.1*
G3: Nottingham combined histologic score 8–9	35	*19*			39	*39*					53%	74	*26.0*
**ER status**									0.004	0.079			
Negative	17	*9*			21	*21*					55%	38	*13.3*
Positive	134	*73*			60	*59*					31%	194	*68.1*
Unknown	33	*18*			20	*20*					38%	53	*18.6*
**PR status**									0.015	0.869			
Negative	35	*19*			31	*31*					47%	66	*23.2*
Positive	116	*63*			50	*50*					30%	166	*58.2*
Unknown	33	*18*			20	*20*					38%	53	*18.6*
**HER2/neu expression**									0.256	NA			
Not overexpressed, 0+ or 1+	81	*44*			38	*38*					32%	119	*41.8*
Equivocal, weak overexpression, 2+	2	*1*			2	*2*					50%	4	*1.4*
Overexpressed, 3+	29	*16*			23	*23*					44%	52	*18.2*
Unknown	72	*39*			38	*38*					35%	110	*38.6*
**Angiolymphatic invasion**									<0.001	<0.001			
None	95	*52*			23	*23*					19%	118	*41.4*
Present	25	*14*			70	*69*					74%	95	*33.3*
Unknown	64	*35*			8	*8*					11%	72	*25.3*
***Sentinel Lymph Node Characteristics***													
**No. SLNs Removed**			1.96	0.07			1.87	0.09	0.511	NA			
= 1	71	*39*			42	*42*			0.806		37%	113	*39.6*
= 2	67	*36*			37	*37*					36%	104	*36.5*
>2	46	*25*			22	*22*					32%	68	*23.9*
**No. SLNs Tumor-involved**			1.33	0.05			1.39	0.07	0.426	NA			
= 1	137	*73*			71	*70*			0.679		34%	208	*73.0*
= 2	38	*21*			23	*23*					38%	61	*21.4*
>2	9	*6*			7	*7*					44%	16	*5.6*
**Size of SLN metastases§**									<0.001	<0.001			
Isolated tumor cells or clusters ≤ 0.2 mm	61	*33*			3	*3*					4.7%	64	*22.5*
Micrometastases, >0.2 mm to 2 mm	117	*64*			83	*82*					42%	200	*70.2*
Macrometastases, >2 mm	6	*3*			15	*15*					71%	21	*7.4*
**Sentinel lymph node metastases identification**									<0.001	NA			
Hematoxylin and eosin staining	122	*61*			98	*97*					45%	220	*77.2*
Immunohistochemistry	62	*34*			3	*3*					4.6%	65	*22.8*

∫ Univariate analyses calculated by χ 2 test and Wilcoxon rank sum test.

∫∫ Multivariate analyses calculated by logistic regression of significant factors by univariate analysis.

*SEM, standard error of the mean.

†Determined according to modified Scarff-Bloom-Richardson grading system.

§Determined according to AJCC criteria, 6th ed.

NA, variable not included in multivariate analysis (not an independent factor or not significant in univariate analysis)

The MSKCC Breast Cancer Nomogram for Prediction of ALN Status [13] (Nomogram) was applied to our patient population and, to provide fair comparison, calculated for only the 171 patients with complete information on the eight variables required for its application (pathologic size of primary tumor, tumor type with nuclear grade if ductal, LVI, multifocality of primary tumor, ER status, method of detecting SLN metastasis, number of positive SLNs, and number of negative SLNs; a ninth variable, whether a frozen section was performed, was not applicable to our patients). ROC curves were constructed for the Nomogram and the other methods to compare the area under the curve (AUC). Internal validation was performed by 10-fold cross-validation, as previously described [27]. Data were divided at random into 10 parts, as equal as possible in size. CART (in this instance, but more generally any other procedure) was then computed successively for 9/10 of the data with the remaining piece held out as "test sample." This was repeated 10 times and results on the 10 test samples were averaged. Cross-validation is an internal validation method that estimates performance on subsequent subjects by eliminating bias that owes to using the same, or even a portion of the same, data for both modeling and testing. However, even with internal validation, bias and variability can be introduced into subsequent analyses if the prevalence of features that predict outcome is different in future datasets than in the dataset from which the model was developed. The differences in distribution of variables (and in synergistic interactions between variables) for an original and a subsequent test dataset impacts a model's performance on future datasets and applies both to our models and to that of the Nomogram. For this reason, we tested our model and the Nomogram on the Northwestern dataset that differed from our original dataset in its distribution of patient, tumor, and sentinel node variables.

ROC curves were constructed for the Nomogram and the MLR informed by CART model for the Bay Area SLN study dataset and the independent Northwestern dataset.

Statistical analyses were performed with R [33].

## Results

Table 1 and Additional files 2 and 3 describe in detail the SLN-negative and SLN-positive patients of the *Bay Area SLN *dataset. As expected, the incidence of SLN metastasis increased with increasing tumor size: 29% of T1, 51% of T2, and 80% of T3 tumors had SLN metastasis. As tumor size increased over 1 cm, the incidence of angiolymphatic invasion doubled for both SLN-positive and SLN-negative patients but was higher for SLN-positive patients (Additional file 3). Among all 784 patients, the total number of women with any axillary lymph node metastasis was 316 (40%), including 31 (9.8%) with a false negative SLN (Additional file 2).

Among SLN-positive cases, the average number of SLNs removed was 1.91, with metastatic disease limited to a single SLN in 73%. Among tumor-involved SLNs, 23% contained isolated tumor cells or clusters (ITCs, ≤ 0.2 mm); 70% contained micrometastases (>0.2 mm to 2 mm); and 7% contained macrometastases (>2 mm). All SLNs containing ITCs required IHC for detection. Only one of 200 cases with SLNs involved by micrometastasis was not observed on H&E and required IHC staining for identification. All 21 cases with SLN macrometastasis were identified by H&E staining (Additional file 2).

**Figure 1:**
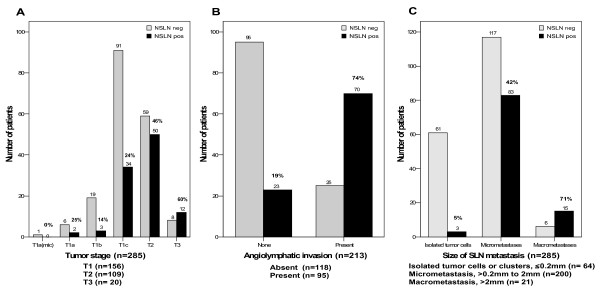
Fraction of patients in Bay Area SLN Database with and without NSLN metastases in relation to (A) tumor stage, (B) angiolymphatic invasion, and (C) size of SLN metastasis.

Of 285 patients with tumor-involved SLNs, 101 (35.4%) were found to have NSLN metastases, with tumor metastases to two or more NSLNs in the majority of cases (median number of positive NSLNs 2; mean 3.5; range 1–19) (Additional file 2). By univariate analyses, 8 variables were highly predictive of NSLN status: tumor size (in cm), tumor size by AJCC T classification, tumor grade, ER status, PR status, angiolymphatic invasion, size of SLN metastasis, and whether the nodal metastasis was identified by H&E or IHC (Table 1). Of patients whose SLN was identified by H&E, 45% had NSLN metastases, whereas only 4.6% of patients whose SLN was identified by IHC had NSLN metastases (*p *< 0.001). Size of SLN metastasis and staining method for metastasis identification are highly correlated (*p *= 0.02, by χ^2 ^testing) and therefore are not independent predictors of NSLN status. Thus, staining method for identifying tumor-involvement was not included in the multivariate analysis shown in Table 1. By multivariate analysis, tumor size, angiolymphatic invasion, and size of SLN metastasis remained significantly predictive of NSLN status (*p *< 0.001 by unconditional testing). Of the 285 patients with SLN metastases, NSLN metastases were found in 25% of patients with T1 tumors; in 46% with T2 tumors; and in 60% with T3 tumors (Figure 1A). When angiolymphatic invasion was present, there was a 3.9-fold increase in NSLN metastases (74% vs. 19%, Figure 1B). Among patients with isolated tumor cells or clusters within the SLN, 4.7% had NSLN metastasis; whereas 42% of patients with micrometastasis and 71% with macrometastasis had NSLN involvement (Figure 1C and Table 1).

**Figure 2:**
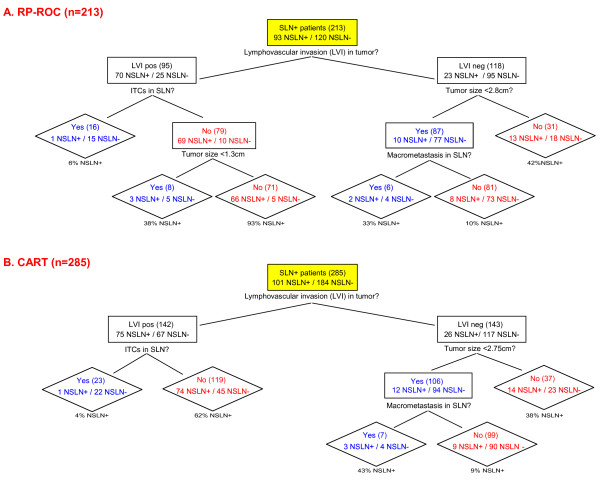
**Tree diagrams for RP-ROC and CART.** As CART is able to impute missing data, it was calculated for all SLN positive patients, n = 285. RP-ROC requires complete data and was calculated for patients with known angiolymphatic invasion status, n = 213 (Bay Area SLN Database).

The models generated by RP-ROC (Figure 2A) and CART (Figure 2B, Additional files 4 and 5) ultimately included tumor size, angiolymphatic invasion, and size of SLN metastasis. At the final split, likelihood of NSLN metastases partitioned into groups by level of risk. The significant predictors as selected by multivariate tree-based modeling were tested individually, as well as all iterations of predictors, in a MLR model. Variables entered were tumor size, angiolymphatic invasion, and size of SLN metastasis (Table 2). Size of SLN metastases interacts with the status of angiolymphatic invasion; that is, the impact of the size of SLN metastases upon the presence or absence of NSLN metastases depends on whether there was angiolymphatic invasion. The tree suggests that one might enter angiolymphatic invasion (scored as 1 if present, 0 if absent) not only multiplied by SLN metastasis size to the first power, but also as the product of angiolymphatic invasion and the square of SLN metastasis size (scored as an ordinal variable with values of 1, 2, and 3 corresponding to the size classification of isolated tumor cells, micrometastasis, or macrometastasis). The MLR model identified two highly predictive composite variables: the product of angiolymphatic invasion and size of SLN metastasis (*p *< 0.0001, odds ratio of 4.73 with approximate 95% confidence interval 3.11–7.20) as well as the product of tumor size and squared size of SLN metastasis (*p *< 0.0001, odds ratio of 1.18 with 95% confidence interval 1.10–1.26). We emphasize that *p*-values are only approximate because CART was used as preprocessor to manufacturing the predictive variables. However, these *p*-values are so small, and the clinical logic so compelling, that we do not doubt their practical, let alone statistical, significance.

**Table 2:** Multivariate Logistic Regression (MLR) analysis informed by CART for predicting NSLN metastasis among SLN+ patients (n = 213) (Bay Area SLN Database).

**Variable or Composite Variable**	**Significance**	**Wald Statistic**	**OR (95% CI)**
Angiolymphatic Invasion	0.024	a	a
Tumor Size	0.508	a	a
Size of SLN Metastasis	0.173	a	a
Angiolymphatic Invasion × Tumor Size	0.166	a	a
Angiolymphatic Invasion × Size of SLN Metastasis	<0.0001	52.7	4.73 (3.11–7.20)
Angiolymphatic Invasion × (Size of SLN Metastasis)^2^	0.041	a	a
Tumor Size × Size of SLN Metastasis	0.888	a	a
Tumor Size × (Size of SLN Metastasis)^2^	<0.0001	22.6	1.18 (1.10–1.26)
Tumor Size × Size of SLN Metastasis × Angiolymphatic Invasion	0.471	a	a
Tumor Size × (Size of SLN Metastasis)^2 ^× Angiolymphatic Invasion	0.761	a	a

OR: odds ratio; 95% CI: 95% confidence interval. ^a ^No Wald statistic or odds ratio calculated for variables not significant at *P *< 0.01 with respect to multivariate model

**Figure 3:**
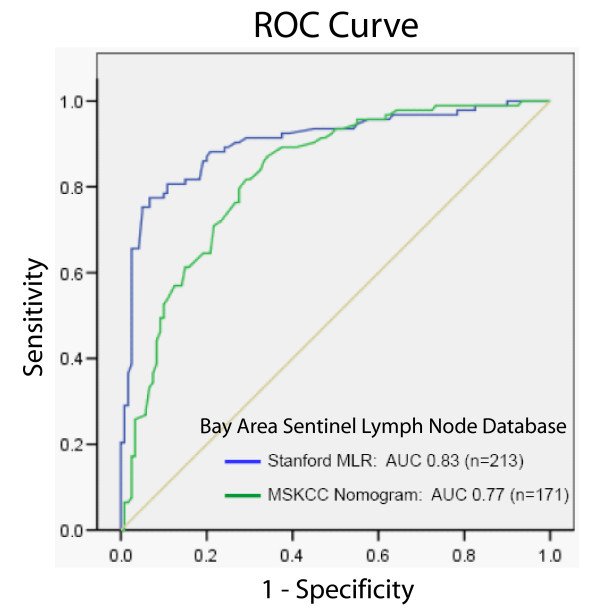
**ROC curves for MLR informed by CART calculation in blue, AUC = 0.83, and Nomogram in green, AUC = 0.77, when applied to the Bay Area SLN Database.** Note that MLR informed by CART calculation was done for larger group of patients (n = 213). When it was performed for the same patient group as the Nomogram (n = 171), AUC increased to 0.85.

Table 3 compares the sensitivities, specificities, and predictive accuracies of our three models, RP-ROC, boosted CART, and MLR, all computed with 10-fold cross validation [26]. As different models require different information, we evaluated models for the entire group (n = 285, only possible for CART) and subsets that contained complete information on angiolymphatic invasion (n = 213), and alternatively, on angiolymphatic invasion and ER status (n = 171). Cross-validated sensitivities/specificities of the three technologies for the group with known angiolymphatic invasion status (n = 213) were 79%/76% for RP-ROC, 88%/71% for boosted CART, and 78%/86% for MLR. Cross-validated specificity of boosted CART when inferred for the entire dataset (n = 285) was lower than when calculated using known values for angiolymphatic invasion (n = 213), suggesting that angiolymphatic invasion is informative in our dataset. This is supported by the continued selection of angiolymphatic invasion in CART modeling when patients have known angiolymphatic invasion status (n = 213) and known angiolymphatic status and ER status (n = 171) (Additional files 4 and 5, respectively). Overall diagnostic accuracy, based on areas under the ROC curve [34] (AUC), for predicting NSLN metastasis among patients in our database was greatest by MLR (83% and 85%) for the subsets of patients for whom the computation was possible (n = 213 and n = 171, respectively). Further, we applied the Nomogram to our SLN-positive patients who had complete data available for entry of its eight variables (n = 171, all patients with known angiolymphatic invasion status and ER status). Figure 3 shows a graph of the ROC curve that devolves from our MLR using our two composite variables (n = 213) and the ROC curve that devolves from the Nomogram (n = 171). Because much preprocessing has gone into our computations, *p*-values we might report (regarding a null hypothesis that the "true" areas under the curves are equal) would be suspect. However, the diagnostic accuracy or area under the curve (AUC) for our MLR is 83% (95% confidence interval 0.81–0.86), and the AUC for the Nomogram is 77% (95% confidence interval 0.73–0.81). When we use the same patients as used in the Nomogram for the MLR calculation (n = 171), our model achieves cross-validated AUC of 85% (95% confidence interval 0.81–0.89). Given that only three variables were used to calculate our MLR, the difference is noteworthy.

**Table 3:** Model comparisons for predicting NSLN metastasis among SLN+ patients (Bay Area SLN Database).

	**For all SLN+ Pts (n = 285)**	**For pts with known angiolymphatic invasion status (n = 213)**	**For pts with known angiolymphatic invasion and ER status (n = 171)**
**RP-ROC with 10-fold cross validation**			
Sensitivity (%)		78.8	83.2
Specificity (%)		75.5	78.1
Diagnostic Accuracy by AUC (%)		**76.7**	**80.2**
			
**Boosted CART with 10-fold cross validation**			
Sensitivity (%)	78.2	87.9	89.0
Specificity (%)	62.0	71.4	74.7
Diagnostic Accuracy by AUC (%)	**67.7**	**77.5**	**80.3**
			
**Multivariable logistic regression with 10-fold cross validation**			
Sensitivity (%)		78.0	78.9
Specificity (%)		86.2	88.3
Diagnostic Accuracy by AUC (%)		**83.3**	**84.9**
			
			
**MSKCC Breast Cancer Nomogram**^a^			
Diagnsotic Accuracy by AUC (%)			**76.7**

^a ^Memorial Sloan-Kettering Cancer Center Breast Cancer Nomogram for Prediction of Axillary Lymph Node Metastasis applied to SLN+ pts in Stanford dataset who have complete data for all Nomogram variables

Finally, the MLR and Nomogram were applied to a database of 77 patients who received ALND for positive SLNs at Northwestern University (Additional file 6). The SLN metastases in this dataset were identified by H&E stain without IHC. Among the 77 SLN positive patients, 61% had T1 tumors, 36% had T2 tumors, and 2.6% had T3 tumors. Angiolymphatic invasion was present in 68% of patients' tumors, and the SLN metastases in the Northwestern dataset were predominantly of large tumor burden with 56% having macrometastasis. NSLN metastases were present in 24 patients (31%). This is in contrast to the Bay Area SLN dataset with 55% T1 tumors, 38% T2 tumors, and 7% T3 tumors; 45% with angiolymphatic invasion (when angiolymphatic invasion status was known); 7% with macrometastasis; and 35% NSLN metastases (Table 1 and Figure 1). Although the Northwestern tumors were somewhat smaller, the higher percentage of angiolymphatic invasion and SLN macrometastases suggest more biologically aggressive disease in their dataset, yet they had a slightly lower percentage of NSLN metastasis.

**Figure 4:**
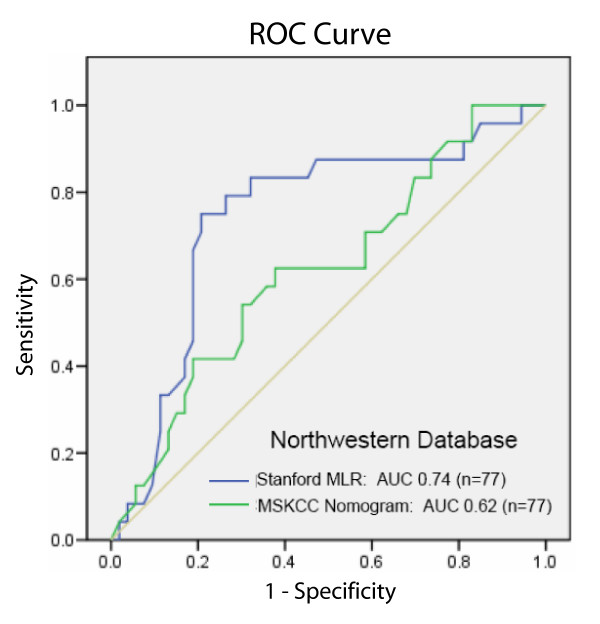
**ROC curves for MLR informed by CART calculation in blue, AUC = 0.74, and Nomogram in green, AUC = 0.62, when applied to the Northwestern test set (n = 77).** 24 patients had NSLN metastasis in this dataset.

Both the MLR model and the Nomogram performed less well when applied to the Northwestern dataset; however, the MLR model was supported with an AUC of 77% (95% confidence interval 0.67–0.80). This is superior to the performance of the Nomogram among this population, 62% (95% confidence interval 0.55–0.68) (Figure 4).

## Discussion

Sentinel lymph node biopsy is a major advance in the treatment of women with breast cancer [35]. If no SLN metastases are identified, the likelihood of additional NSLN involvement is 9.8% in our series. Though above the goal false-negative rate proposed by the American Society of Breast Surgeons, this is comparable to that reported in NSABP-32 and recently by both Lyman and Veronesi ranging 9.7%, 8.4%, and 8.8% respectively [1,36,37]. Of our patients with positive SLNs, the majority presented with micrometastasis, 70%, or isolated tumor cells, 22%. Thus, our population contains a predominance of limited SLN disease burden relative to prior reports, including van Rijk's reported rate of 23% for micrometastasis and 16% for isolated tumor cells [38]. This may be important as suggested by Alran et al. who showed lower performance of the Nomogram in patients with only micrometastases [19]. Despite the seemingly low sentinel node tumor burden, 35% had NSLN metastases upon completion ALND. Unfortunately, no combination of clinical and/or pathologic characteristics enabled identification of all SLN-positive patients at risk for NSLN metastases. Although SLN-positive patients will receive systemic chemotherapy and/or hormone therapy, it is unknown whether occult NSLN metastases are eradicated by adjuvant treatment. Until results of large prospective clinical trials can demonstrate no long-term increase in mortality from omitting ALND in the setting of systemic therapy, prophylactic ALND for patients with tumor-involved SLNs, including those with and without NSLN involvement, remains standard surgical care [39-43]. However, in practice, it is the patient and her physician who decide whether or not a completion axillary dissection is performed. This decision may be informed using online calculators such as the Nomogram and the one presented here.

Based on a multi-institutional sample set larger than most prior studies, we found that univariate predictors of NSLN status include tumor size (in cm and by AJCC T size classification), tumor grade, hormone receptor status (ER and PR), angiolymphatic invasion, size of SLN metastasis, and whether nodal tumor involvement is identified by H&E. By multivariate analyses, tumor size, angiolymphatic invasion, size of SLN metastases, and products of these variables predict NSLN tumor involvement. Others have also discovered the predictive strength of each of the three simple characteristics [5,6,10,44-50], although here we confirm their collective power in a unique way. Additionally, we found that angiolymphatic invasion is as strong a predictor of NSLN metastasis as is size of SLN metastasis.

For women with isolated tumor cells in the SLN, we found a 4.7% chance of NSLN involvement, similar to Calhoun and Giuliano's reported NSLN-involvement rate of 4.9% for the same subset of patients, and comparable to or lower than that found previously, 10–15% [47,51,52]. The benefits of no further axillary dissection must be weighed against the risk of harboring axillary metastasis that may potentially seed occult metastatic disease. Clinical context, with consideration of a patient's expected life-span and associated health problems, may impact the definition of a "minimal acceptable risk." Recommendations for clinical practice are difficult because the risk of NSLN metastasis in SLN-positive patients with isolated tumor cells is comparable or lower than the risk of NSLN metastases in patients without SLN metastases (9.8% in our study) [53]. These issues are being studied in large-scale prospective clinical trials [40,41]. Future molecular technologies may also provide guidance [54,55].

Our goal was to identify patients with tumor-free NSLNs who, with near certainty, may be spared completion ALND. Using multivariate tree-based modeling by RP-ROC, boosted CART, and MLR informed by CART, we identified tumor size, angiolymphatic invasion, and size of SLN metastasis as characteristics that optimized stratification of NSLN status. These refined, statistical analyses demonstrated a highly synergistic interaction between size of SLN metastasis and angiolymphatic invasion on risk of NSLN metastasis. Our models (Figures 2, Additional files 4 and 5) stratified patients with tumor-involved SLNs into four risk groups for having NSLN metastasis: low risk (10% or less), moderate risk (30–45%), high risk (about 60%), and very high risk (greater than 90%).

MLR modeling of NSLN status that was informed by CART in its selection of predictors provided the most accurate cross-validated technique for predicting NSLN metastases for patients with known angiolymphatic invasive status, with accuracy superior to boosted CART, RP-ROC, and the Memorial Sloan-Kettering Breast Cancer Nomogram. When applied to the Bay Area SLN Database, the Nomogram had an AUC of 77%. This compares with the accuracy of the Nomogram for the original MSKCC population (76%) and for a prospective cohort at MSKCC (77%) [13]. Seven subsequent studies have tested the Nomogram and show an accuracy of 63% to 86%, though as low as 54% when applied only to patients with SLN micrometastases [14,17-19,56-60]. In contrast, our MLR informed by CART model performed equally well among patients with isolated tumor cells, micrometastases, or macrometastases. Relative importance of size of SLN metastasis in the Nomogram is determined by method of detection including IHC, serial H&E, routine analysis, versus frozen section (among the subset for which this is performed) [13]. The improved predictive accuracy of the MLR model informed by CART, particularly among patients with isolated tumor cells or micrometastasis, may be due to the relative weight ascribed to the specific size of SLN metastasis in our model. Application of our MLR model to other patient populations is required to validate its performance.

Considering the risk of potential bias due to low sentinel node tumor burden in our dataset, we applied both our model and the MSKCC Nomogram to an independent dataset of 77 SLN positive patients who underwent completion ALND. These cases were not identified by IHC and this dataset contained cases with a much larger tumor burden: 56% of cases contained macrometastasis in the SLN compared to 7% of the Bay Area SLN dataset. Again, the MLR model showed superior performance to the Nomogram. However, the performance of both models decreased compared to the Bay Area SLN Database: the Stanford Online Calculator generated an AUC of 0.74, or 74%, and the Nomogram generated an AUC of 0.62, or 62%. This raises concern regarding the generalizable nature of any model. An underlying reason why neither model performed as well as anticipated is that when a model is developed based on data from one group of patients, and the model is subsequently applied to data from a different group of patients, performance is generally diminished [24,27]. This is due to differences in the distributions of predictive features and differences in the synergistic impact (interactions) between and among these features in different groups of patients. Thus a model developed in one group of patients would not be expected to perform as well for a different group of patients, even if the performance of the model was validated internally (cross-validated) on the original group. Table 1 and Additional file 6 shows differences in the distribution of the three variables in our model – tumor size, size of SLN metastasis, and angiolymphatic invasion – between patients in the Bay Area SLN Database and Northwestern series. As we would also expect the interactions of these variables to be different for both groups, we believe these factors in aggregate may be responsible for our findings.

The predictive accuracy of the Nomogram requires assessment of eight tumor characteristics [13,14,17,18,56]. A hazard of multi-variable modeling is that its overall accuracy is dependent upon the accuracy and precision with which each individual variable is determined. Our MLR confirmed the importance of two composite variables from only three tumor characteristics: 1) the size of SLN metastasis when angiolymphatic invasion is present, and 2) tumor size times the square of the size of SLN metastasis. The first composite variable reflects the synergism between angiolymphatic invasion and size of SLN metastasis; the second involves tumor burden. Using these two composite variables, AUC is 83% or 85% compared to the Nomogram's AUC of 77% that relies on eight variables. By using statistical methods which allow assessment of the variable-variable interactions we demonstrate superior accuracy with fewer required variables. Our model is the first proposed which emphasizes the synergistic interactions among patient characteristics. By reducing the required variables, we are hopeful the MLR model may be applied a larger population of patients, without excluding those with incomplete, unavailable, or pending pathologic data.

Missing pathologic data is problematic for breast cancer patients nationwide. Though the generalizability of our model may have benefited from the diverse population represented, obtaining complete clinicopathologic information was partially limited by enrollment across 16 institutions during the years of our study, 1996–2002. Approximately 25% of our 285 SLN-positive patients had no histologic analysis for angiolymphatic invasion. Of the 213 patients with angiolymphatic data present, another 19.7% had no ER status performed or recorded. This is comparable to the 17.1% of invasive breast cancer patients without recorded ER status in 13 registries of the national Surveillance, Epidemiology, and End Results (SEER) database from 1999–2003 [61] (unpublished data, Jeffrey lab); presence of angiolymphatic invasion status was not requested by SEER. Thus, we analyzed our data using three patient groups: the entire SLN-positive dataset of 285 patients; 213 SLN-positive patients who had complete information on angiolymphatic invasion; and 171 SLN-positive patients who had complete information on angiolymphatic invasion and ER status. Even applying the smallest dataset, more SLN-positive patients are analyzed than in most other published studies.

Though not directly compared among identical patient populations, the AUC of our model is also superior to that of M.D. Anderson Cancer Center scoring system (70%) and the Hôpital Tenon scoring system derived in Paris, France (68%), as recently reported by Dauphine *et al. *[59,62,63]. Calculations using our MLR model are easily done over the internet with the Stanford Online Calculator [64]. We encourage others to access and test our model and directly compare it with other models for evaluating risk of NSLN metastasis.

Although no modeling technique has been able to identify patients without any risk of NSLN metastasis, Park *et al*. recently argued that ALND may be reasonably eliminated among patients with approximately 9% or less predicted risk of NSLN involvement [16]. A low risk subset of 287 patients with SLN metastasis were followed in a non-randomized study with a 2% observed rate of local recurrence. This recommendation, however, is limited by a follow-up of only 23 months. We expect that data from two large prospective clinical studies, NSABP-32 and American College of Surgeons trial Z0011, will more definitively resolve questions regarding the optimal surgical management of SLN-positive patients [40,41]. In the meantime, we hope that our calculator may provide further guidance for risk evaluation.

## Conclusion

Fewer than half of women undergoing completion axillary lymph node dissection (ALND) for breast cancer will have non-sentinel node (NSLN) metastasis. We present a new model and the Stanford Online Calculator developed from a Northern California and Oregon database with superior accuracy and simplicity (three versus eight required patient variables) compared to the Memorial Sloan-Kettering Breast Cancer Nomogram for our dataset and another independent dataset. We hope that other institutions will test our model using their datasets, which will contain different patient demographics, to validate its accuracy and to refine in which populations it may be best used. Further investigation of predictive models to stratify risk of non-sentinel lymph node metastasis will better define their role in guiding clinical decision-making, while we await the results of larger randomized trials.

## Abbreviations

ALND, axillary lymph node dissection; LN, lymph node; SLN, sentinel lymph node; NSLN, non-sentinel lymph node; RP-ROC, recursive partitioning with receiver operating characteristic curves; CART, boosted classification and regression trees; MLR, multivariate logistic regression; MSKCC, Memorial Sloan-Kettering Cancer Center; Nomogram, MSKCC Breast Cancer Nomogram; ER, estrogen receptor; PR, progesterone receptor; AUC, area under ROC curve; LVI, lymphovascular invasion; H&E, hematoxylin and eosin; IHC, immunohistochemistry; ITC, isolated tumor cells.

## Competing interests

Richard A. Olshen is one of the original developers of CART^®^, the software for which is distributed by Salford Systems of San Diego, CA. The remaining authors have no competing interests to declare.

## Authors' contributions

HEK, RAO, and SSJ conceived the study, performed data analysis, and drafted the manuscript with critical input from RWC, FMD, WHG, DLJ, RVR, FES, and ILW. LB, RWC, FMD, JJD, WHG, SSJ, DLJ, VJP, and FES developed the Bay Area SLN Study Database and assisted in patient accrual, clinical data acquisition, or data review. RVR processed and analyzed sentinel node tissue blocks. DJW and SH wrote the software for the Stanford Online Calculator and created the website. HRB obtained IRB approval and compiled all Northwestern SLN test data under the guidance of NMH. All authors read and approved the final manuscript.

## Pre-publication history

The pre-publication history for this paper can be accessed here:



## Supplementary Material

Additional file 1 Schematic of patients accrued to Bay Area SLN Database. An overview of the entire Bay Area SLN Database and exclusion criteria for this study. Click here for file

Additional file 2 Patient, primary tumor, and lymph node characteristics among SLN-negative and SLN-positive patients from the Bay Area SLN Database. This table describes the demographics of the patient, primary tumor, and lymph node characteristics among SLN-negative and SLN-positive patients from the Bay Area SLN Database. Click here for file

Additional file 3 The relationship of angiolymphatic invasion and size of SLN metastasis to tumor size (Bay Area SLN Database). This table shows A. the occurrence of angiolymphatic invasion with increasing tumor size for SLN-negative and SLN-positive patients, and B. size of SLN metastasis with increasing tumor size (Bay Area SLN Database). Click here for file

Additional file 4 CART decision tree for patients with complete data on angiolymphatic invasion status, n = 213 (Bay Area SLN Database). The figure shows the CART decision tree for 213 patients with complete data on angiolymphatic invasion status. Click here for file

Additional file 5 CART decision tree for patients with complete data on angiolymphatic invasion status and ER status, n = 171 (Bay Area SLN Database). The figure shows the CART decision tree for patients with complete data on angiolymphatic invasion status and ER status. Click here for file

Additional file 6 Patient, primary tumor, and lymph node characteristics among SLN-positive and SLN-negative patients from the Northwestern dataset. The table describes the demographics of the patient, primary tumor, and lymph node characteristics among SLN-negative and SLN-positive patients from the Northwestern dataset. Click here for file

## References

[B1] Lyman GH, Giuliano AE, Somerfield MR, Benson AB, Bodurka DC, Burstein HJ, Cochran AJ, Cody HS, Edge SB, Galper S, Hayman JA, Kim TY, Perkins CL, Podoloff DA, Sivasubramaniam VH, Turner RR, Wahl R, Weaver DL, Wolff AC, Winer EP (2005). American Society of Clinical Oncology guideline recommendations for sentinel lymph node biopsy in early-stage breast cancer. J Clin Oncol.

[B2] Rubio IT, Korourian S, Cowan C, Krag DN, Colvert M, Klimberg VS (1998). Sentinel lymph node biopsy for staging breast cancer. Am J Surg.

[B3] Carlson RW, McCormick B (2005). Update: NCCN breast cancer Clinical Practice Guidelines. J Natl Compr Canc Netw.

[B4] Sosa JA, Diener-West M, Gusev Y, Choti MA, Lange JR, Dooley WC, Zeiger MA (1998). Association between extent of axillary lymph node dissection and survival in patients with stage I breast cancer. Ann Surg Oncol.

[B5] Chu KU, Turner RR, Hansen NM, Brennan MB, Bilchik A, Giuliano AE (1999). Do all patients with sentinel node metastasis from breast carcinoma need complete axillary node dissection?. Ann Surg.

[B6] Turner RR, Chu KU, Qi K, Botnick LE, Hansen NM, Glass EC, Giuliano AE (2000). Pathologic features associated with nonsentinel lymph node metastases in patients with metastatic breast carcinoma in a sentinel lymph node. Cancer.

[B7] Rietman JS, Dijkstra PU, Geertzen JH, Baas P, De Vries J, Dolsma W, Groothoff JW, Eisma WH, Hoekstra HJ (2003). Short-term morbidity of the upper limb after sentinel lymph node biopsy or axillary lymph node dissection for Stage I or II breast carcinoma. Cancer.

[B8] Hack TF, Cohen L, Katz J, Robson LS, Goss P (1999). Physical and psychological morbidity after axillary lymph node dissection for breast cancer. J Clin Oncol.

[B9] Mansel RE, Fallowfield L, Kissin M, Goyal A, Newcombe RG, Dixon JM, Yiangou C, Horgan K, Bundred N, Monypenny I, England D, Sibbering M, Abdullah TI, Barr L, Chetty U, Sinnett DH, Fleissig A, Clarke D, Ell PJ (2006). Randomized multicenter trial of sentinel node biopsy versus standard axillary treatment in operable breast cancer: the ALMANAC Trial. J Natl Cancer Inst.

[B10] Reynolds C, Mick R, Donohue JH, Grant CS, Farley DR, Callans LS, Orel SG, Keeney GL, Lawton TJ, Czerniecki BJ (1999). Sentinel lymph node biopsy with metastasis: can axillary dissection be avoided in some patients with breast cancer?. J Clin Oncol.

[B11] Abdessalam SF, Zervos EE, Prasad M, Farrar WB, Yee LD, Walker MJ, Carson WB, Burak WE (2001). Predictors of positive axillary lymph nodes after sentinel lymph node biopsy in breast cancer. Am J Surg.

[B12] Rahusen FD, Torrenga H, van Diest PJ, Pijpers R, van der Wall E, Licht J, Meijer S (2001). Predictive factors for metastatic involvement of nonsentinel nodes in patients with breast cancer. Arch Surg.

[B13] Van Zee KJ, Manasseh DM, Bevilacqua JL, Boolbol SK, Fey JV, Tan LK, Borgen PI, Cody HS, Kattan MW (2003). A nomogram for predicting the likelihood of additional nodal metastases in breast cancer patients with a positive sentinel node biopsy. Ann Surg Oncol.

[B14] Smidt ML, Kuster DM, van der Wilt GJ, Thunnissen FB, Van Zee KJ, Strobbe LJ (2005). Can the Memorial Sloan-Kettering Cancer Center Nomogram predict the likelihood of nonsentinel lymph node metastases in breast cancer patients in the Netherlands?. Ann Surg Oncol.

[B15] Specht MC, Kattan MW, Gonen M, Fey J, Van Zee KJ (2005). Predicting nonsentinel node status after positive sentinel lymph biopsy for breast cancer: clinicians versus nomogram. Ann Surg Oncol.

[B16] Park J, Fey JV, Naik AM, Borgen PI, Van Zee KJ, Cody HS (2007). A declining rate of completion axillary dissection in sentinel lymph node-positive breast cancer patients is associated with the use of a multivariate nomogram. Ann Surg.

[B17] Kocsis L, Svebis M, Boross G, Sinko M, Maraz R, Rajtar M, Cserni G (2004). Use and limitations of a nomogram predicting the likelihood of non-sentinel node involvement after a positive sentinel node biopsy in breast cancer patients. Am J Surg.

[B18] Degnim AC, Reynolds C, Pantvaidya G, Zakaria S, Hoskin T, Barnes S, Roberts MV, Lucas PC, Oh K, Koker M, Sabel MS, Newman LA (2005). Nonsentinel node metastasis in breast cancer patients: assessment of an existing and a new predictive nomogram. Am J Surg.

[B19] Alran S, De Rycke Y, Fourchotte V, Charitansky H, Laki F, Falcou MC, Benamor M, Freneaux P, Salmon RJ, Sigal-Zifrani B (2007). Validation and limitations of use of a breast cancer nomogram predicting the likelihood of non-sentinel node involvement after positive sentinel node biopsy. Ann Surg Oncol.

[B20] Cody HS, Borgen PI (1999). State-of-the-art approaches to sentinel node biopsy for breast cancer: study design, patient selection, technique, and quality control at Memorial Sloan-Kettering Cancer Center. Surg Oncol.

[B21] Cserni G, Amendoeira I, Apostolikas N, Bellocq JP, Bianchi S, Bussolati G, Boecker W, Borisch B, Connolly CE, Decker T, Dervan P, Drijkoningen M, Ellis IO, Elston CW, Eusebi V, Faverly D, Heikkila P, Holland R, Kerner H, Kulka J, Jacquemier J, Lacerda M, Martinez-Penuela J, De Miguel C, Peterse JL, Rank F, Regitnig P, Reiner A, Sapino A, Sigal-Zafrani B, Tanous AM, Thorstenson S, Zozaya E, Wells CA (2003). Pathological work-up of sentinel lymph nodes in breast cancer. Review of current data to be considered for the formulation of guidelines. Eur J Cancer.

[B22] Elston CW, Ellis IO (1991). Pathological prognostic factors in breast cancer. I. The value of histological grade in breast cancer: experience from a large study with long-term follow-up. Histopathology.

[B23] Singletary SE, Greene FL, Sobin LH (2003). Classification of isolated tumor cells: clarification of the 6th edition of the American Joint Committee on Cancer Staging Manual. Cancer.

[B24] Breiman L, Friedman JH, Olshen RA, Stone CJ (1984). Classification and Regression Trees.

[B25] Dalgaard P (2002). Introductory Statistics with R.

[B26] Kraemer HC (1992). Evaluating medical tests: objective and quantitative guidelines.

[B27] Hastie T, Tibshirani R, Friedman JH (2001). The Elements of Statistical Learning.

[B28] Kwak LW, Halpern J, Olshen RA, Horning SJ (1990). Prognostic significance of actual dose intensity in diffuse large-cell lymphoma: results of a tree-structured survival analysis. J Clin Oncol.

[B29] ROC software, Sierra-Pacific MIRECC. http://mirecc.stanford.edu/.

[B30] CART software, Salford Systems. http://www.salford-systems.com/cart.php.

[B31] McLachlan G (1992). Discriminant Analysis and Statistical Pattern Recognition.

[B32] Stone CJ (1995). A Course in Probability and Statistics.

[B33] Team RDC (2006). R: A Language and Environment for Statistical Computing..

[B34] DeLong ER, DeLong DM, Clarke-Pearson DL (1988). Comparing the areas under two or more correlated receiver operating characteristic curves: a nonparametric approach. Biometrics.

[B35] Veronesi U, Paganelli G, Viale G, Luini A, Zurrida S, Galimberti V, Intra M, Veronesi P, Robertson C, Maisonneuve P, Renne G, De Cicco C, De Lucia F, Gennari R (2003). A randomized comparison of sentinel-node biopsy with routine axillary dissection in breast cancer. N Engl J Med.

[B36] Esserman L, Sepucha K (2005). Practice implications of the high false negative rate of sentinel lymph node biopsy reported in NSABP-32. J Clin Oncol.

[B37] Veronesi U, Paganelli G, Viale G, Luini A, Zurrida S, Galimberti V, Intra M, Veronesi P, Maisonneuve P, Gatti G, Mazzarol G, De Cicco C, Manfredi G, Fernandez JR (2006). Sentinel-lymph-node biopsy as a staging procedure in breast cancer: update of a randomised controlled study. Lancet Onc.

[B38] van Rijk MC, Peterse JL, Nieweg OE, Oldenburg HS, Rutgers EJ, Kroon BB (2006). Additional axillary metastases and stage migration in breast cancer patients with micrometastases or submicrometastases in sentinel lymph nodes. Cancer.

[B39] Orr RK (1999). The impact of prophylactic axillary node dissection on breast cancer survival--a Bayesian meta-analysis. Ann Surg Oncol.

[B40] Krag DN, Julian TB, Harlow SP, Weaver DL, Ashikaga T, Bryant J, Single RM, Wolmark N (2004). NSABP-32: Phase III, randomized trial comparing axillary resection with sentinal lymph node dissection: a description of the trial. Ann Surg Oncol.

[B41] Grube BJ, Giuliano AE (2001). Observation of the breast cancer patient with a tumor-positive sentinel node: implications of the ACOSOG Z0011 trial. Semin Surg Oncol.

[B42] Fant JS, Grant MD, Knox SM, Livingston SA, Ridl K, Jones RC, Kuhn JA (2003). Preliminary outcome analysis in patients with breast cancer and a positive sentinel lymph node who declined axillary dissection. Ann Surg Oncol.

[B43] Wada N, Imoto S, Yamauchi C, Hasebe T, Ochiai A (2006). Predictors of tumour involvement in remaining axillary lymph nodes of breast cancer patients with positive sentinel lymph node. Eur J Surg Oncol.

[B44] Kamath VJ, Giuliano R, Dauway EL, Cantor A, Berman C, Ku NN, Cox CE, Reintgen DS (2001). Characteristics of the sentinel lymph node in breast cancer predict further involvement of higher-echelon nodes in the axilla: a study to evaluate the need for complete axillary lymph node dissection. Arch Surg.

[B45] Yu JC, Hsu GC, Hsieh CB, Sheu LF, Chao TY (2005). Prediction of metastasis to non-sentinel nodes by sentinel node status and primary tumor characteristics in primary breast cancer in Taiwan. World J Surg.

[B46] Viale G, Maiorano E, Pruneri G, Mastropasqua MG, Valentini S, Galimberti V, Zurrida S, Maisonneuve P, Paganelli G, Mazzarol G (2005). Predicting the risk for additional axillary metastases in patients with breast carcinoma and positive sentinel lymph node biopsy. Ann Surg.

[B47] Bolster MJ, Peer PGM, Bult P, Thunnissen FBJM, Schapers RFM, Meijer JWR, Strobbe LJA, van Berlo CLH, Klinkenbijl JHG, Beex LVAM, Wobbes T, Tjan-Heijnen VCG (2007). Risk factors for non-sentinel lymph node metastases in patients with breast cancer. The outcome of a multi-institutional study. Ann Surg Oncol.

[B48] Carcoforo P, Maestroni U, Querzoli P, Lanzara S, Maravegias K, Feggi L, Soliani G, Basaglia E (2006). Primary breast cancer features can predict additional lymph node involvement in patients with sentinel node micrometastases. World J Surg.

[B49] Rivers AK, Griffith KA, Hunt KK, Degnim AC, Sabel MS, Diehl KM, Cimmino VM, Chang AE, Lucas PC, Newman LA (2006). Clinicopathologic features associated with having four or more metastatic axillary nodes in breast cancer patients with a positive sentinel lymph node. Ann Surg Oncol.

[B50] Ozmen V, Karanlik H, Cabioglu N, Igci A, Kecer M, Asoglu O, Tuzlali S, Mudun A (2006). Factors predicting the sentinel and non-sentinel lymph node metastases in breast cancer. Breast Cancer Res Treat.

[B51] Calhoun KE, Hansen NM, Turner RR, Giuliano AE (2005). Nonsentinel node metastases in breast cancer patients with isolated tumor cells in the sentinel node: implications for completion axillary node dissection. Am J Surg.

[B52] Cserni G, Gregori D, Merletti F, Sapino A, Mano MP, Ponti A, Sandrucci S, Baltas B, Bussolati G (2004). Meta-analysis of non-sentinel node metastases associated with micrometastatic sentinel nodes in breast cancer. Br J Surg.

[B53] Cserni G, Bianchi S, Vezzosi V, Arisio R, Bori R, Peterse JL, Sapino A, Castellano I, Drijkoningen M, Kulka J, Eusebi V, Foschini MP, Bellocq JP, Marin C, Thorstenson S, Amendoeira I, Reiner-Concin A, Decker T, Lacerda M, Figueiredo P, Fejes G (2007). Sentinel lymph node biopsy in staging small (up to 15 mm) breast carcinomas. Results from a European multi-institutional study. Path Onc Res.

[B54] Kohrt HE, Nouri N, Nowels K, Johnson D, Holmes S, Lee PP (2005). Profile of immune cells in axillary lymph nodes predicts disease-free survival in breast cancer. PLoS Med.

[B55] Jeffrey SS, Lonning PE, Hillner BE (2005). Genomics-based prognosis and therapeutic prediction in breast cancer. J Natl Compr Canc Netw.

[B56] Soni NK, Carmalt HL, Gillett DJ, Spillane AJ (2005). Evaluation of a breast cancer nomogram for prediction of non-sentinel lymph node positivity. Eur J Surg Oncol.

[B57] Lambert LA, Ayers GD, Hwang RF, Hunt KK, Ross MI, Kuerer HM, Singletary SE, Babiera GV, Ames FC, Feig B, Lucci A, Krishnamurthy S, Meric-Bernstam F (2006). Validation of a breast cancer nomogram for predicting nonsentinel lymph node metastases after a positive sentinel node biopsy. Ann Surg Oncol.

[B58] Cripe MH, Beran LC, Liang WC, Sickle-Santanello BJ (2006). The likelihood of additional nodal disease following a positive sentinel lymph node biopsy in breast cancer patients: validation of a nomogram. Am J Surg.

[B59] Dauphine CE, Haukoos JS, Vargas MP, Isaac NM, Khalkhali I, Vargas HI (2007). Evaluation of three scoring systems predicting non sentinel node metastasis in breast cancer patients with a positive sentinel node biopsy. Ann Surg Oncol.

[B60] Ponzone R, Maggiorotto F, Mariani L, Jacomuzzi ME, Magistris A, Mininanni P, Biglia N, Sismondi P (2007). Comparison of two models for the prediction of nonsentinel node metastases in breast cancer. Am J Surg.

[B61] Surveillance Epidemiology and End Results Statistics. http://seer.cancer.gov/.

[B62] Hwang RF, Krishnamurthy S, Hunt KK, Mirza N, Ames FC, Feig B, Kuerer HM, Singletary SE, Babiera G, Meric F, Akins JS, Neely J, Ross MI (2003). Clinicopathologic factors predicting involvement of nonsentinel axillary nodes in women with breast cancer. Ann Surg Oncol.

[B63] Barranger E, Coutant C, Flahault A, Delpech Y, Darai E, Uzan S (2005). An axilla scoring system to predict non-sentinel lymph node status in breast cancer patients with sentinel lymph node involvement. Breast Cancer Res Treat.

[B64] Stanford Online Calculator. http://www-stat.stanford.edu/~olshen/NSLNcalculator/.

