# Supplementary material for: New models and online calculator for predicting non-sentinel lymph node status in sentinel lymph node positive breast cancer patients
Source: BMC Cancer. 2008 Mar 4;8:66. doi: 10.1186/1471-2407-8-66 (PMC2311316; doi:10.1186/1471-2407-8-66)
Supplement: Additional file 1 — Schematic of patients accrued to Bay Area SLN Database. An overview of the entire Bay Area SLN Database and exclusion criteria for this study. [file 1471-2407-8-66-S1.pdf]

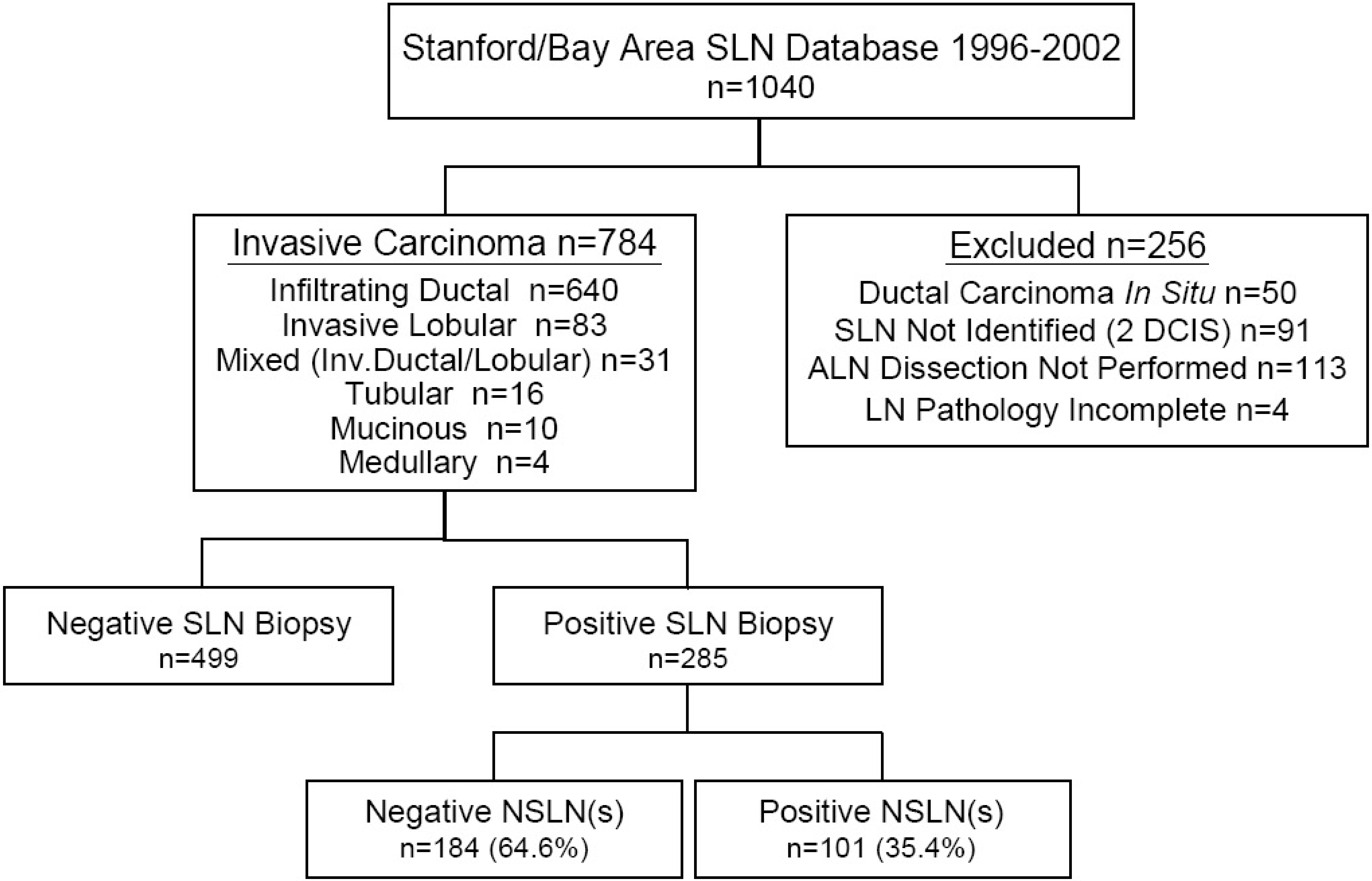
Stanford/Bay Area SLN Database 1996-2002
n=1040
Invasive Carcinoma n=784
Infiltrating Ductal n=640
Invasive Lobular n=83
Mixed (Inv.Ductal/Lobular) n=31
Tubular n=16
Mucinous n=10
Medullary n=4
Excluded n=256
Ductal Carcinoma *In Situ* n=50
SLN Not Identified (2 DCIS) n=91
ALN Dissection Not Performed n=113
LN Pathology Incomplete n=4
Negative SLN Biopsy
n=499
Positive SLN Biopsy
n=285
Negative NSLN(s)
n=184 (64.6%)
Positive NSLN(s)
n=101 (35.4%)
