# Supplementary material for: New models and online calculator for predicting non-sentinel lymph node status in sentinel lymph node positive breast cancer patients
Source: BMC Cancer. 2008 Mar 4;8:66. doi: 10.1186/1471-2407-8-66 (PMC2311316; doi:10.1186/1471-2407-8-66)
Supplement: Additional file 2 — Patient, primary tumor, and lymph node characteristics among SLN-negative and SLN-positive patients from the Bay Area SLN Database. This table describes the demographics of the patient, primary tumor, and lymph node characteristics among SLN-negative and SLN-positive patients from the Bay Area SLN Database. [file 1471-2407-8-66-S2.doc]

|  | **Tumor-free SLN (SLN-)** | | | | | **Tumor-involved SLN (SLN+)** | | | | | **% SLN+** (SLN+/Total) | **TOTAL** | |
| --- | --- | --- | --- | --- | --- | --- | --- | --- | --- | --- | --- | --- | --- |
| ***Patient and Tumor Characteristics*** | Number of Pts (n=499) | *%* | Mean | Median | Range | Number of Pts (n=285) | *%* | Mean | Median | Range |  | Number of Pts (n=784) | *%* |
| **Patient Age** (years) |  |  | 57 | 56 | 25-85 |  |  | 55 | 53 | 28 - 84 |  |  |  |
| **Tumor Type** |  |  |  |  |  |  |  |  |  |  |  |  |  |
| Infiltrating Ductal Carcinoma | 394 | *79.0* |  |  |  | 246 | *86.3* |  |  |  | *38.4* | 640 | *81.6* |
| Invasive Lobular Carcinoma | 56 | *11.2* |  |  |  | 27 | *9.5* |  |  |  | *32.5* | 83 | *10.6* |
| Mixed Carcinoma | 20 | *4.0* |  |  |  | 11 | *3.9* |  |  |  | *35.5* | 31 | *4.0* |
| Tubular Carcinoma | 15 | *3.0* |  |  |  | 1 | *0.4* |  |  |  | *6.3* | 16 | *2.0* |
| Mucinous Carcinoma | 10 | *2.0* |  |  |  | 0 | *0.0* |  |  |  | *0.0* | 10 | *1.3* |
| Medullary Carcinoma | 4 | *0.8* |  |  |  | 0 | *0.0* |  |  |  | *0.0* | 4 | *0.5* |
| **Tumor size (cm)** |  |  | 1.65 | 1.60 | 0.1-14.7 |  |  | 2.41 | 2 | 0.1 - 12.0 |  |  |  |
| **Tumor size (AJCC)** |  |  |  |  |  |  |  |  |  |  |  |  |  |
| T1 | 390 | *78.2* |  |  |  | 156 | *54.7* |  |  |  | *28.6* | 546 | *69.6* |
| T1a (mic) | 1 | *0.2* |  |  |  | 1 | *0.4* |  |  |  | *50.0* | 2 | *0.3* |
| T1a | 28 | *5.6* |  |  |  | 8 | *2.8* |  |  |  | *22.2* | 36 | *4.6* |
| T1b | 114 | *22.8* |  |  |  | 22 | *7.7* |  |  |  | *16.2* | 136 | *17.3* |
| T1c | 247 | *49.5* |  |  |  | 125 | *43.9* |  |  |  | *33.6* | 372 | *47.4* |
| T2 | 104 | *20.8* |  |  |  | 109 | *38.2* |  |  |  | *51.2* | 213 | *27.2* |
| T3 | 5 | *1.0* |  |  |  | 20 | *7.0* |  |  |  | *80.0* | 25 | *3.2* |
| **Tumor grade†** |  |  |  |  |  |  |  |  |  |  |  |  |  |
| G1: Nottingham combined score 3-5 | 486 | *97.4* |  |  |  | 91 | *31.9* |  |  |  | *15.8* | 577 | *73.6* |
| G2: Nottingham combined score 6-7 | 7 | *1.4* |  |  |  | 120 | *42.1* |  |  |  | *94.5* | 127 | *16.2* |
| G3: Nottingham combined score 8-9 | 6 | *1.2* |  |  |  | 74 | *26.0* |  |  |  | *92.5* | 80 | *10.2* |
| **ER status** |  |  |  |  |  |  |  |  |  |  |  |  |  |
| Negative | 64 | *12.8* |  |  |  | 38 | *13.3* |  |  |  | *37.3* | 102 | *13.0* |
| Positive | 334 | *66.9* |  |  |  | 194 | *68.1* |  |  |  | *36.7* | 528 | *67.3* |
| Unknown | 101 | *20.2* |  |  |  | 53 | *18.6* |  |  |  | *34.4* | 154 | *19.6* |
| **PR status** |  |  |  |  |  |  |  |  |  |  |  |  |  |
| Negative | 123 | *24.6* |  |  |  | 66 | *23.2* |  |  |  | *34.9* | 189 | *24.1* |
| Positive | 274 | *54.9* |  |  |  | 166 | *58.2* |  |  |  | *37.7* | 440 | *56.1* |
| Unknown | 102 | *20.4* |  |  |  | 53 | *18.6* |  |  |  | *34.2* | 155 | *19.8* |
| **HER2/neu expression** |  |  |  |  |  |  |  |  |  |  |  |  |  |
| Not overexpressed, 0+ or 1+ | 190 | *38.1* |  |  |  | 119 | *41.8* |  |  |  | *38.5* | 309 | *39.4* |
| Equivocal, weak overexpression, 2+ | 20 | *4.0* |  |  |  | 4 | *1.4* |  |  |  | *16.7* | 24 | *3.1* |
| Overexpressed, 3+ | 34 | *6.8* |  |  |  | 52 | *18.2* |  |  |  | *60.5* | 86 | *11.0* |
| Unknown | 255 | *51.1* |  |  |  | 110 | *38.6* |  |  |  | *30.1* | 365 | *46.6* |
| **Angiolymphatic invasion** |  |  |  |  |  |  |  |  |  |  |  |  |  |
| None | 310 | *62.1* |  |  |  | 118 | *41.4* |  |  |  | *27.6* | 428 | *54.6* |
| Present | 48 | *9.6* |  |  |  | 95 | *33.3* |  |  |  | *66.4* | 143 | *18.2* |
| Unknown | 141 | *28.3* |  |  |  | 72 | *25.3* |  |  |  | *33.8* | 213 | *27.2* |
|  |  |  |  |  |  |  |  |  |  |  |  |  |  |
| ***Sentinel Lymph Node Characteristics*** |  |  |  |  |  |  |  |  |  |  |  |  |  |
| **No. SLNs Removed** |  |  | 1.81 | 1 | 1.0-8.0 |  |  | 1.91 | 2 | 1.0 - 5.0 |  |  |  |
| =1 | 272 | *54.5* |  |  |  | 113 | *39.6* |  |  |  | *29.4* | 385 | *49.1* |
| =2 | 126 | *25.3* |  |  |  | 104 | *36.5* |  |  |  | *45.2* | 230 | *29.3* |
| >2 | 101 | *20.2* |  |  |  | 68 | *23.9* |  |  |  | *40.2* | 169 | *21.6* |
| **No. SLNs Tumor-involved** |  |  | 0 | 0 | 0 |  |  | 1.35 | 1 | 1.0 - 4.0 |  |  |  |
| =0 | 499 | *100.0* |  |  |  | 0 | *0.0* |  |  |  |  | 499 | *63.6* |
| =1 | 0 | *0.0* |  |  |  | 208 | *73.0* |  |  |  |  | 208 | *26.5* |
| =2 | 0 | *0.0* |  |  |  | 61 | *21.4* |  |  |  |  | 61 | *7.8* |
| >2 | 0 | *0.0* |  |  |  | 16 | *5.6* |  |  |  |  | 16 | *2.0* |
| **Size and identification of SLN metastases§** |  |  |  |  |  |  |  |  |  |  |  |  |  |
| Isolated tumor cells or clusters ≤0.2mm |  |  |  |  |  | 64 | *22.5* |  |  |  |  |  |  |
| *Identified by hematoxylin and eosin staining* |  |  |  |  |  | 0 | *0.0* |  |  |  |  |  |  |
| *Upgraded by immunohistochemistry* |  |  |  |  |  | 64 | *100.0* |  |  |  |  |  |  |
| Micrometastases, >0.2mm to 2mm |  |  |  |  |  | 200 | *70.2* |  |  |  |  |  |  |
| Identified by hematoxylin and eosin staining |  |  |  |  |  | 199 | *99.5* |  |  |  |  |  |  |
| Upgraded by immunohistochemistry |  |  |  |  |  | 1 | *0.5* |  |  |  |  |  |  |
| Macrometastases, >2mm |  |  |  |  |  | 21 | *7.4* |  |  |  |  |  |  |
| Identified by hematoxylin and eosin staining |  |  |  |  |  | 21 | *100.0* |  |  |  |  |  |  |
| Upgraded by immunohistochemistry |  |  |  |  |  | 0 (N/A) | *0.0* |  |  |  |  |  |  |
| **Sentinel lymph node metastases identification method** | | |  |  |  |  |  |  |  |  |  |  |  |
| Hematoxylin and eosin staining |  |  |  |  |  | 220 | *77.2* |  |  |  |  |  |  |
| Immunohistochemistry |  |  |  |  |  | 65 | *22.8* |  |  |  |  |  |  |
|  |  |  |  |  |  |  |  |  |  |  |  |  |  |
| ***Axillary Lymph Node Characteristics*** |  |  |  |  |  |  |  |  |  |  |  |  |  |
| **No. NSLNs Removed in ALN dissection** |  |  |  |  |  |  |  | 10.6 | 10 | 1.0 - 32.0 |  |  |  |
| ≤10 | 276 | *55.3* |  |  |  | 126 | *44.2* |  |  |  |  |  |  |
| >10 | 223 | *44.7* |  |  |  | 159 | *55.8* |  |  |  |  |  |  |
| **No. NSLNs Tumor-involved** |  |  |  |  |  |  |  | 3.47 | 2 | 1.0 - 19.0 |  |  |  |
| ≥1 | 31 | *6.2* |  |  |  | 101 | *100.0* |  |  |  |  |  |  |
| =1 | 13 | *41.9* |  |  |  | 34 | *33.7* |  |  |  |  |  |  |
| =2 | 9 | *29.0* |  |  |  | 25 | *24.8* |  |  |  |  |  |  |
| =3 | 6 | *19.3* |  |  |  | 11 | *10.9* |  |  |  |  |  |  |
| =4 | 0 | *0.0* |  |  |  | 11 | *10.9* |  |  |  |  |  |  |
| >4 | 3 | *9.7* |  |  |  | 20 | *19.8* |  |  |  |  |  |  |
