# Supplementary material for: New models and online calculator for predicting non-sentinel lymph node status in sentinel lymph node positive breast cancer patients
Source: BMC Cancer. 2008 Mar 4;8:66. doi: 10.1186/1471-2407-8-66 (PMC2311316; doi:10.1186/1471-2407-8-66)
Supplement: Additional file 3 — The relationship of angiolymphatic invasion and size of SLN metastasis to tumor size (Bay Area SLN Database). This table shows A. the occurrence of angiolymphatic invasion with increasing tumor size for SLN-negative and SLN-positive patients, and B. size of SLN metastasis with increasing tumor size (Bay Area SLN Database). [file 1471-2407-8-66-S3.doc]

|  | | | **Primary Tumor Size - T Stage** | | | | | | | | | | | |  | | | |
| --- | --- | --- | --- | --- | --- | --- | --- | --- | --- | --- | --- | --- | --- | --- | --- | --- | --- | --- |
|  | All Patients,  n=784 | | T1a (mic) ≤0.1cm, n=2 | | T1a ≤0.5cm, n=36 | | T1b ≤1.0cm, n=136 | | T1c ≤2cm, n=372 | | T2 ≤5.0cm, n=213 | | T3 >5.0cm, n=25 | | Pts with known angiolymphatic invasion status, n=571 | | Pts with known angiolymphatic invasion status and ER status,  n=453 | |
| **A. SLN Status & Angiolymphatic Invasion** | **#** | *%* | # | *%* | # | *%* | # | *%* | # | *%* | # | % | # | % | # | % | # | % |
| **Tumor-involved SLN** | **285** | ***36.4*** | **1** | ***50.0*** | **8** | ***22.2*** | **22** | ***16.2*** | **125** | ***33.6*** | **109** | ***51.2*** | **20** | ***80.0*** | **213** | ***37.3*** | **171** | ***37.7*** |
| No angiolymphatic invasion | 118 | *41.4* | 1 | *100.0* | 6 | *75.0* | 11 | *50.0* | 58 | *46.4* | 34 | *31.2* | 8 | *40.0* | 118 | *55.4* | 93 | *54.4* |
| Angiolymphatic invasion present | 95 | *33.3* | 0 | *0.0* | 1 | *12.5* | 3 | *13.6* | 37 | *29.6* | 46 | *42.2* | 8 | *40.0* | 95 | *44.6* | 78 | *45.6* |
| Unknown | 72 | *25.3* | 0 | *0.0* | 1 | *12.5* | 8 | *36.4* | 30 | *24.0* | 29 | *26.6* | 4 | *20.0* |  |  |  |  |
| **Tumor-free SLN** | **499** | ***63.6*** | **1** | ***50.0*** | **28** | ***77.8*** | **114** | ***83.8*** | **247** | ***66.4*** | **104** | ***48.8*** | **5** | ***20.0*** | **358** | ***62.7*** | **282** | ***62.3*** |
| No angiolymphatic invasion | 310 | *62.1* | 0 | *0.0* | 20 | *71.4* | 77 | *67.5* | 149 | *60.3* | 60 | *57.7* | 4 | *80.0* | 310 | *86.6* | 245 | *86.9* |
| Angiolymphatic invasion present | 48 | *9.6* | 1 | *100.0* | 0 | *0.0* | 5 | *4.4* | 23 | *9.3* | 18 | *17.3* | 1 | *20.0* | 48 | *13.4* | 37 | *13.1* |
| Unknown | 141 | *28.3* | 0 | *0.0* | 8 | *28.6* | 32 | *28.1* | 75 | *30.4* | 26 | *25.0* | 0 | *0.0* |  |  |  |  |
|  |  |  |  |  |  |  |  |  |  |  |  |  |  |  |  |  |  |  |
| **B. SLN Status & SLN Metastasis Size** |  |  |  |  |  |  |  |  |  |  |  |  |  |  |  |  |  |  |
| **Tumor-involved SLN** | **285** | ***36.4*** | **1** | ***50.0*** | **8** | ***22.2*** | **22** | ***16.2*** | **125** | ***33.6*** | **109** | ***51.2*** | **20** | ***80.0*** | **213** | ***37.3*** | **171** | ***37.7*** |
| Isolated tumor cells or clusters, ≤0.2mm | 64 | *22.5* | 1 | *100.0* | 4 | *50.0* | 7 | *31.8* | 28 | *22.4* | 18 | *16.5* | 6 | *30.0* | 50 | *23.5* | 39 | *22.8* |
| Micrometastasis, >0.2mm to 2mm | 200 | *70.2* | 0 | *0.0* | 4 | *50.0* | 14 | *63.6* | 86 | *68.8* | 82 | *75.2* | 14 | *70.0* | 145 | *68.1* | 119 | *69.6* |
| Macrometastasis, >2mm | 21 | *7.4* | 0 | *0.0* | 0 | *0.0* | 1 | *4.5* | 11 | *8.8* | 9 | *8.3* | 0 | *0.0* | 18 | *8.5* | 13 | *7.6* |
