# Supplementary figures and images for: New models and online calculator for predicting non-sentinel lymph node status in sentinel lymph node positive breast cancer patients
Source: BMC Cancer. 2008 Mar 4;8:66. doi: 10.1186/1471-2407-8-66 (PMC2311316; doi:10.1186/1471-2407-8-66)

## CART (n=213)

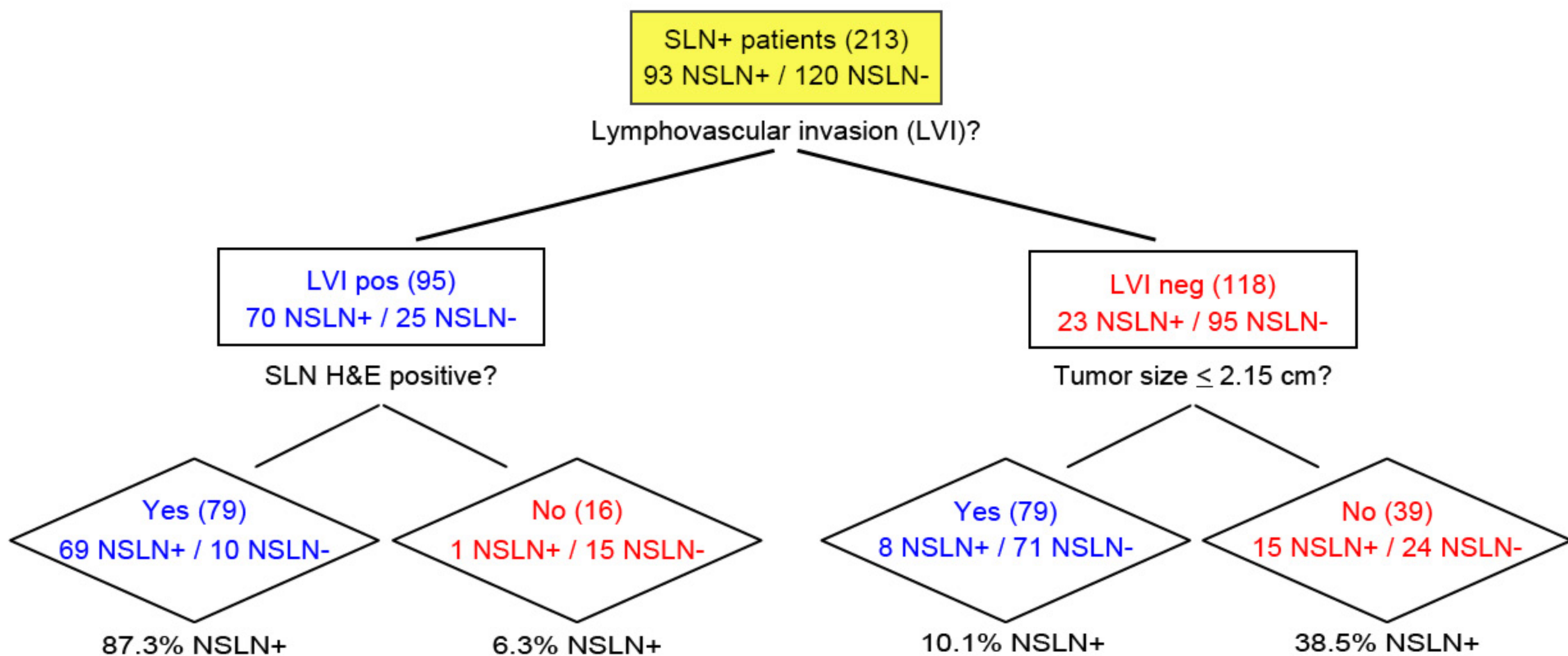

Supplement: Additional file 4 — CART decision tree for patients with complete data on angiolymphatic invasion status, n = 213 (Bay Area SLN Database). The figure shows the CART decision tree for 213 patients with complete data on angiolymphatic invasion status. [file 1471-2407-8-66-S4.pdf]

# CART (n=171)

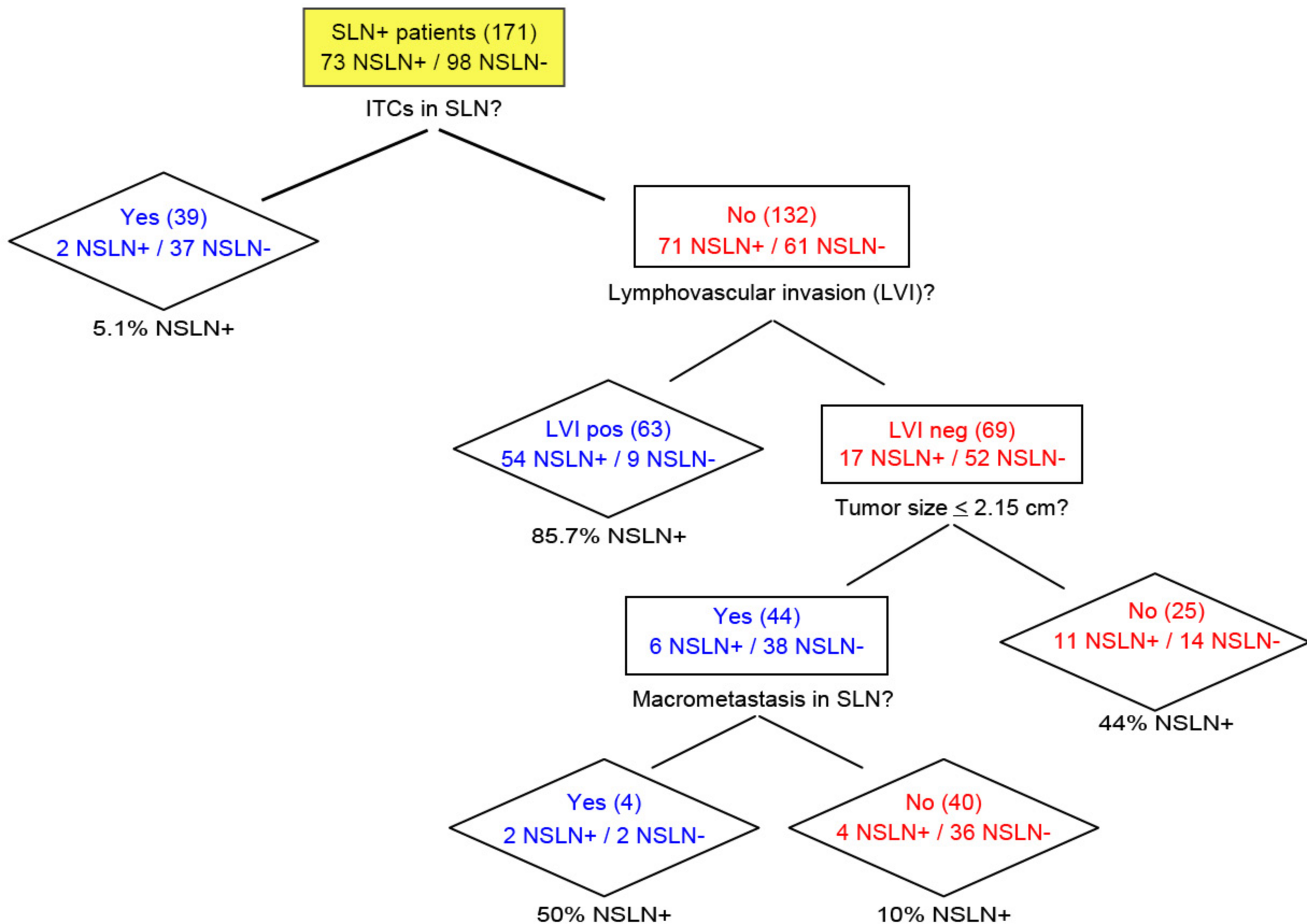

Supplement: Additional file 5 — CART decision tree for patients with complete data on angiolymphatic invasion status and ER status, n = 171 (Bay Area SLN Database). The figure shows the CART decision tree for patients with complete data on angiolymphatic invasion status and ER status. [file 1471-2407-8-66-S5.pdf]
