# Supplementary material for: New models and online calculator for predicting non-sentinel lymph node status in sentinel lymph node positive breast cancer patients
Source: BMC Cancer. 2008 Mar 4;8:66. doi: 10.1186/1471-2407-8-66 (PMC2311316; doi:10.1186/1471-2407-8-66)
Supplement: Additional file 6 — Patient, primary tumor, and lymph node characteristics among SLN-positive and SLN-negative patients from the Northwestern dataset. The table describes the demographics of the patient, primary tumor, and lymph node characteristics among SLN-negative and SLN-positive patients from the Northwestern dataset. [file 1471-2407-8-66-S6.doc]

|  | **Tumor-free NSLN** | | | | **Tumor-involved NSLN** | | | |
| --- | --- | --- | --- | --- | --- | --- | --- | --- |
| **Patient and Tumor Characteristics** | Number of Patients (n=53) | *%* | Mean | SEM* | Number of Patients (n=24) | *%* | Mean | SEM* |
| **Patient Age (years)** |  |  | 50.8 | 1.48 |  |  | 48.3 | 1.73 |
| **Tumor Type** |  |  |  |  |  |  |  |  |
| Infiltrating Ductal Carcinoma | 37 | *70* |  |  | 17 | *71* |  |  |
| Invasive Lobular Carcinoma | 5 | *9* |  |  | 0 | *0* |  |  |
| Mixed Carcinoma | 10 | *19* |  |  | 7 | *29* |  |  |
| Tubular Carcinoma | 1 | *2* |  |  | 0 | *0* |  |  |
| **Tumor size (cm)** |  |  | 2.03 | 0.2 |  |  | 2.36 | 0.28 |
| **Tumor size (AJCC)** |  |  |  |  |  |  |  |  |
| T1 | 33 | *62* |  |  | 14 | *58* |  |  |
| T1a(mic) | 1 | *2* |  |  | 0 | *0* |  |  |
| T1a | 1 | *2* |  |  | 1 | *4* |  |  |
| T1b | 6 | *11* |  |  | 1 | *4* |  |  |
| T1c | 25 | *47* |  |  | 12 | *50* |  |  |
| T2 | 19 | *36* |  |  | 9 | *38* |  |  |
| T3 | 1 | *2* |  |  | 1 | *4* |  |  |
| **Tumor grade†** |  |  |  |  |  |  |  |  |
| G1: Nottingham combined histologic score 3-5 | 15 | *28* |  |  | 5 | *21* |  |  |
| G2: Nottingham combined histologic score 6-7 | 20 | *38* |  |  | 14 | *58* |  |  |
| G3: Nottingham combined histologic score 8-9 | 18 | *34* |  |  | 5 | *21* |  |  |
| **ER status** |  |  |  |  |  |  |  |  |
| Negative | 9 | *17* |  |  | 4 | *17* |  |  |
| Positive | 44 | *83* |  |  | 20 | *83* |  |  |
| Unknown | 0 | *0* |  |  | 0 | *0* |  |  |
| **PR status** |  |  |  |  |  |  |  |  |
| Negative | 18 | *34* |  |  | 8 | *33* |  |  |
| Positive | 35 | *66* |  |  | 16 | *67* |  |  |
| Unknown | 0 | *0* |  |  | 0 | *0* |  |  |
| **HER2/neu expression** |  |  |  |  |  |  |  |  |
| Not overexpressed, 0+ or 1+ | 47 | *89* |  |  | 18 | *75* |  |  |
| Equivocal, weak overexpression, 2+ | 2 | *4* |  |  | 0 | *0* |  |  |
| Overexpressed, 3+ | 4 | *7* |  |  | 6 | *25* |  |  |
| Unknown | 0 | *0* |  |  | 0 | *0* |  |  |
| **Angiolymphatic invasion** |  |  |  |  |  |  |  |  |
| None | 21 | *40* |  |  | 4 | *17* |  |  |
| Present | 32 | *60* |  |  | 20 | *83* |  |  |
| Unknown | 0 | *0* |  |  | 0 | *0* |  |  |
| **Sentinel Lymph Node Characteristics** |  |  |  |  |  |  |  |  |
| **No. SLNs Removed** |  |  | 2.51 | 0.18 |  |  | 2.38 | 0.24 |
| =1 | 15 | *28* |  |  | 5 | *21* |  |  |
| =2 | 13 | *25* |  |  | 10 | *42* |  |  |
| >2 | 25 | *48* |  |  | 9 | *37* |  |  |
| **No. SLNs Tumor-involved** |  |  | 1.3 | 0.18 |  |  | 1.63 | 0.19 |
| =1 | 40 | *75* |  |  | 15 | *63* |  |  |
| =2 | 11 | *21* |  |  | 7 | *29* |  |  |
| >2 | 2 | *4* |  |  | 2 | *8* |  |  |
| **Size of SLN metastases§** |  |  |  |  |  |  |  |  |
| Isolated tumor cells or clusters ≤0.2mm | 2 | *4* |  |  | 0 | *0* |  |  |
| Micrometastases, >0.2mm to 2mm | 27 | *51* |  |  | 5 | *21* |  |  |
| Macrometastases, >2mm | 24 | *45* |  |  | 19 | *79* |  |  |
| **Sentinel lymph node metastases identification** |  |  |  |  |  |  |  |  |
| Hematoxylin and eosin staining** | 53 | *100* |  |  | 24 | *100* |  |  |
| Immunohistochemistry | 0 | *0* |  |  | 0 | *0* |  |  |

*SEM, standard error of the mean

†Determined according to modified Scarff-Bloom-Richardson grading system

§Determined according to AJCC criteria, 6th ed.

**Only serial H&E staining performed by Northwestern pathology
